# P18 Synergistic enhancement of chlorhexidine activity by Polyols against polymicrobial oral biofilms

**DOI:** 10.1093/jacamr/dlag102.024

**Published:** 2026-06-26

**Authors:** Hero Mohammed, Ayesha Rahman, Malgorzata Wiench

**Affiliations:** School of Dentistry, University of Birmingham, Birmingham, UK; School of Dentistry, Institute of Clinical Sciences, University of Birmingham, Birmingham, UK; Institute of Cancer and Genomic Sciences, University of Birmingham, Birmingham, UK

## Abstract

**Background:**

Biofilm-associated antimicrobial tolerance is increasingly recognized as a driver of treatment failure and a contributor to antimicrobial resistance (AMR). Oral polymicrobial biofilms, implicated in dental caries and periodontal disease, exhibit intrinsic tolerance to antiseptics through extracellular polymeric substance (EPS) matrix protection, metabolic heterogeneity, and adaptive stress responses. Chlorhexidine Di gluconate (CHX) remains the clinical gold standard oral antiseptic; however, escalating concerns regarding reduced susceptibility, ecological dysbiosis, and dose-dependent cytotoxicity highlight the need for optimization strategies. This study evaluated whether polyols (xylitol, maltitol, and sorbitol) can act as adjuvants to enhance CHX efficacy against complex cross-kingdom oral biofilms.

**Methods:**

A clinically relevant four-species consortium comprising *Streptococcus mutans, Streptococcus oralis, Streptococcus sanguinis,* and *Candida albicans* was used to model polymicrobial oral biofilms. Planktonic susceptibility testing was performed via standardized broth microdilution assays. Drug–drug interactions were quantified using chequerboard analysis and fractional inhibitory concentration index (FICI) calculations. Biofilms were cultivated under basal, sucrose-enriched (cariogenic), and artificial saliva (physiological simulation) conditions. Biomass was quantified using crystal violet staining, metabolic activity assessed via XTT reduction assay, and oxidative stress measured using ROS-Glo™ hydrogen peroxide detection. Structural and architectural changes were characterized using confocal laser scanning microscopy (CLSM) and scanning electron microscopy (SEM).

**Results:**

CHX demonstrated potent planktonic activity (∼90% inhibition at 4 mg/L), whereas polyols alone required higher concentrations (0.8 g/mL) to achieve comparable effects. Chequerboard analysis demonstrated formulation-dependent synergistic interactions (FICI ≤0.5) between CHX and polyols. Sucrose significantly enhanced biofilm maturation, increasing thickness from 227 ± 8 μm to 353 ± 10 μm. In established biofilms, CHX monotherapy reduced biomass by 80–85%, while combination treatment achieved enhanced suppression (87–90%) and reduced metabolic activity to near-background levels. Combination exposure significantly increased extracellular hydrogen peroxide production, suggesting oxidative stress–mediated disruption. Microscopy confirmed substantial EPS degradation, membrane perturbation, and architectural collapse, reducing biofilm thickness to 16–30 μm.

**Conclusions:**

Polyols significantly potentiate CHX-mediated antibiofilm activity in a clinically relevant polymicrobial oral model. Enhancing antiseptic efficacy at established therapeutic concentrations may reduce the need for higher-dose exposure and limit biofilm-associated tolerance. Optimization of existing antiseptics through synergistic adjuvant strategies represents a pragmatic, stewardship-aligned approach to mitigate AMR risk while improving infection control in oral healthcare settings.

